# Perioperative care of an adolescent with postural orthostatic tachycardia syndrome

**DOI:** 10.4103/1658-354X.62611

**Published:** 2010

**Authors:** Scott Kernan, Joseph D Tobias

**Affiliations:** University of Missouri, School of Medicine University of Missouri, Columbia, Missouri, USA; 1 Departments of Anesthesiology and Pediatrics, University of Missouri, Columbia, Missouri, USA

**Keywords:** *Dysautonomia*, *postural orthostatic tachycardia syndrome*, *POTS*

## Abstract

Postural orthostatic tachycardia syndrome (POTS) is a disorder characterized by postural tachycardia in combination with orthostatic symptoms without associated hypotension. Symptoms include light-headedness, palpitations, fatigue, confusion, and anxiety, which are brought on by assuming the upright position and usually relieved by sitting or lying down. Given the associated autonomic dysfunction that occurs with POTS, various perioperative concerns must be considered when providing anesthetic care for such patients. We present an adolescent with POTS who required anesthetic care during posterior spinal fusion for the treatment of scoliosis. The potential perioperative implications of this syndrome are discussed.

## INTRODUCTION

Postural orthostatic tachycardia syndrome (POTS) is a disorder characterized by postural tachycardia in combination with orthostatic symptoms without associated hypotension. Symptoms include light-headedness, palpitations, fatigue, confusion, and anxiety, which are brought on by assuming the upright position and usually relieved by sitting or lying down.[1‐3] Although possibly described as early as the mid-19^th^ century,[4] the term POTS was first coined by Rosen and Cryer[5] in 1982 with reference to a patient with diabetic neuropathy with a greater than 44 beat/minute increase in heart rate upon standing. An additional description of the syndrome and an outline of the clinical signs and symptoms were subsequently reported by Schondorf and Low in 1993.[6] Various other names have been given to this syndrome including chronic orthostatic intolerance, hyperadrenergic orthostatic tachycardia, familial orthostatic tachycardia, and orthostatic intolerance syndrome.[7] The diagnosis is made based on the development of the classic signs and symptoms and by the demonstration of orthostatic intolerance associated with heart rate changes when assuming the upright position Alternatively, tilt-table testing can be used to further confirm the diagnosis. Confirmatory heart rate changes include either a greater than 30 beat per minute increase above baseline or a heart rate greater than 120 beats per minute within the first 10 minutes of standing or upright tilt.[4]

The syndrome should not be confused with orthostatic hypotension syndromes in which there is a decrease in blood pressure related to venous pooling when assuming the upright position. Given the associated cardiovascular manifestations and the potential for autonomic dysfunction that occurs with POTS,[6] various perioperative concerns must be addressed when providing anesthetic care for such patients. We present an adolescent with POTS who required anesthetic care during posterior spinal fusion for the treatment of scoliosis. The potential anesthetic implications of this syndrome are discussed.

## CASE REPORT

Review of this patient′s medical records and presentation of this case report were approved by the Institutional Review Board of the University of Missouri. A 17-year-old girl with scoliosis, weighing 43 kilograms, presented for posterior spinal fusion with instrumentation from T_4_ to L_4_. Baseline vital signs included blood pressure 113/67 mmHg, heart rate 58 beats/minute, temperature 36.2°C, and oxygen saturation by pulse oximetry of 100% on room air. Her past medical history was significant for POTS, depression, and chronic fatigue. The patient′s symptoms started following a two month period with daily fevers up to 102°F and had been present for approximately four years prior to diagnosis. Initial signs and symptoms included dizzy spells associated with visual changes, joint pain, chest pain, nausea, abdominal pain, and headaches. Further work-up at that time included a C reactive protein level, erythrocyte sedimentation rate, and ANA cascade, all of which were within the normal range. The diagnosis of POTS was made based on postural tachycardia with normotension and tilt-table testing at 12 years of age. Tilt-table testing reproduced her clinical symptoms including light-headedness, palpitations, and nausea. There was a 40-46 beat/minute increase in the heart rate without associated orthostatic hypotension. Despite medical therapy, the patient continued to experience the above mentioned signs and symptoms along with chronic fatigue up to the point of the current surgical procedure. The chronic fatigue limited her activities of daily life to the point that she could participate in only a half day of school. Current medical treatment included midodrine (10 mg three times a day) and metoprolol (12.5 mg twice a day).

The patient was admitted the day of the procedure. She was held *nil* per os for six hours and transferred to the operating room where standard American Society of Anesthesiologists monitors were placed. Inhalational induction was carried out with sevoflurane in nitrous oxide and oxygen. Following anesthetic induction, two peripheral intravenous 16-gauge cannulae were placed. Endotracheal intubation was facilitated with cis-atracurium (0.1 mg/kg). A 5-French, 15 cm, double-lumen, central venous catheter was placed in the right internal jugular vein under ultrasound guidance and a 20 gauge catheter placed in the left radial artery. In an attempt to minimize intraoperative heart rate changes, the CVP which was initially 4-5 mmHg, was increased to and maintained at 8-10 mmHg with the administration of isotonic fluids and colloids. As neurologic monitoring with both motor and somatosensory evoked potentials was planned, maintenance anesthesia consisted of propofol (75-125 μg/kg/min) and remifentanil (0.1-0.25 μg/kg/min). The propofol dose was titrated to maintain the bispectral index at 40-60 while the remifentanil dose was adjusted to maintain the mean arterial pressure (MAP) at 50-65 mmHg. Strategies to limit intraoperative blood loss included controlled hypotension (MAP of 50-65 mmHg) and the administration of the antifibrinolytic agent, epsilon amino caproic acid (5 grams over 30 minutes followed by 1 gram per hour). The patient was turned prone on a Jackson orthopedic operating room table. Intraoperative normothermia was maintained by the use of a forced air warming device and fluid warmers. Intraoperatively, occasional episodes of tachycardia with a heart rate up to 130 beats/minute were treated first with esmolol (10 mg) and then with intermittent bolus doses of metoprolol (1-2 mg as needed – total dose of 5 mg for the case) and occasional bouts of hypotension (MAP less than 50 mmHg) were easily corrected by the administration of bolus doses of phenylephrine (50 μg).

The surgical procedure lasted 8.5 hours. Arterial blood gases, hemoglobin concentration, platelet count, and coagulation function, and electrolytes (sodium, potassium, and ionized calcium) were checked every 1-2 hours throughout the case. The estimated blood loss was 2300 mL. Fluid and colloid administration included 3900 mL of isotonic crystalloid, 850 mL of hetastarch, and 1000 mL of 5% albumin. Blood product administration included three units of packed red blood cells to maintain a hemoglobin concentration ≥ 8 gm/dL, two units of fresh frozen plasma and five units of cryoprecipitate to correct abnormalities in coagulation function and fibrinogen concentration. At the completion of the procedure, the patient′s trachea was extubated. Her postoperative course, both in the Pediatric ICU and the inpatient ward, was uneventful.

## DISCUSSION

Primary POTS comprises a heterogeneous group of disorders that has classically been divided into two main subtypes: (1) the partial dysautonomic or neurogenic form and (2) the hyperadrenergic form.[4] However, it is generally accepted that there is significant overlap between the subtypes in regards to clinical signs and symptoms and therefore such classification may not be clinically applicable or useful. The most common form of POTS is the dysautonomic subtype, occurring in up to 90% of affected patients. In dysautonomic POTS, there is what has been described as a "patchy" dysautonomia leading to an inability of the peripheral vasculature to constrict, which results in pooling of blood in the splanchnic and dependent circulations when the patient assumes the upright position. This results in the reflex activation of the cardiac sympathetic nervous system resulting in the classic finding of tachycardia.[4,8] The exact mechanisms accounting for dysautonomia have been not been full elucidated although immune-mediated and genetic abnormalities have been proposed. Postulated etiologies include peripheral autonomic denervation, an alteration of the baseline sympathetic to parasympathetic balance, and a length-dependent autonomic neuropathy.[7‐9] The hyperadrenergic form of POTS has been attributed to a β-adrenergic receptor hypersensitivity, increased norepinephrine release, or a deficiency in clearance of norepinephrine secondary to a mutation in a reuptake norepinephrine transporter.[4,7] These patients, in contrast to patients with the dysautonomic form of POTS, have elevated serum catecholamine levels, especially during upright posture and an exaggerated response to β-adrenergic agonists.

A third form of POTS has been described in the literature as developmental POTS. This form is primarily seen in adolescents beginning at 12-14 years of age. The cause of this form remains unclear, but the leading hypothesis includes a temporary autonomic imbalance secondary to rapid growth in some adolescents. Developmental POTS produces severe symptoms, peaks around 16 years of age, with symptoms slowing resolving so that up to 80% of patients are asymptomatic by young adulthood.[10,11]

The treatment of primary POTS consists of both conservative treatment and pharmacologic agents. Specific treatment measures include stopping or avoidance of medications which can exacerbate the condition [Table 1], reconditioning with aerobic activity and resistance training of the lower extremities, two liters of fluid intake daily, two to four grams of salt intake daily (unless suffering from hyperadrenergic POTS), compression stockings, and elevation of the head of the bed.[10,12] When conservative treatment fails, pharmacological intervention is warranted. Pharmacological treatment of POTS varies by subtype. Partial dysautonomic POTS is best treated by increasing the peripheral vascular resistance and fluid volume.[10] This is often accomplished with fludrocortisones which promotes sodium and fluid retention, and midodrine which stimulates α_1_ -adrenergic receptors resulting in vasoconstriction. Additionally, some have suggested the use of serotonin selective reuptake inhibitors or norepinephrine reuptake inhibitors (bupropion, duloxetine, or venlafaxine).[10] However, such therapy is not universally accepted given the potential presence of defects in norepinephrine transport protein deficiency as a primary pathophysiologic cause of POTS.[7] Additionally, once the primary pathophysiologic defect (vasodilatation and relative hypovolemia) is corrected, β-adrenergic antagonists may be used to primarily treat the reflex tachycardia. In our patient, pharmacologic therapy include both midodrine to treat the loss of vascular tone and metoprolol to prevent reflex tachycardia.[4,11]

To date, there is only one other report regarding the perioperative care of a patient with POTS undergoing general anesthesia. Mchaourab *et al*. reported their experience with the anesthetic care of a 40-year-old woman with POTS undergoing bilateral mastectomy and breast reconstruction with prostheses.[13] Although no perioperative issues were noted, they stressed the importance of perioperative intravascular volume expansion guided by invasive monitoring and closely monitored controlled of vascular tone with α_1_ -adrenergic receptor agonists (phenylephrine, norepinephrine, and midodrine) to reduce autonomic lability during and after surgery. Their case was complicated by a history suggestive of opioid allergy.

**Table 1:** Medications that can worsen orthostatic intolerance with POTS

Angiotensin converting enzyme inhibitors
Alpha adrenergic antagonists
Angiotensin receptor blocking agents
Bromocriptine
Calcium channel antagonists
Diuretics
Ethanol
Ganglionic blocking agents
Hydralazine
Monoamine oxidase inhibitors
Nitrates
Opiates
Phenothiazines
Sildenafil citrate
Tricyclic antidepressants

Obstetrical patients with POTS undergoing regional anesthesia have also been reported. In separate reports, McEvoy *et al*.[14] Corbett *et al*.[15] and Jones *et al*.[16] described parturients with POTS. These authors all noted the unique challenges of maintaining hemodynamic stability in the obstetrical patient with POTS. They described a need for early pain control to avoid excessive β-adrenergic hyperactivity and suggested the use of epidural rather than spinal anesthesia given the potential for more pronounced and rapid hemodynamic changes with spinal anesthesia. They also noted that the management of the second stage of labor was complicated by increased hemodynamic instability in POTS patients in response to a Valsalva maneuver. More recently, Kanjwal *et al*. reviewed the peripartum outcomes of pregnancy, labor and delivery in 22 women with POTS.[17] Migraine headaches were the most common co-morbid features reported in 41% of the patients. During pregnancy, symptoms of POTS remained unchanged in 3 (13%), improved in 12 (55%), and worsened in seven (31%). All the patients completed pregnancy successfully without an increased incidence of fetal complications or demise.

When considering the overall perioperative care of such patients, there may be specific concerns regarding the preoperative, intraopeative, and postoperative care of such patients. In addition to the primary disease process, several disorders with potential implications during the perioperative stage have been associated with POTS including mitral valve prolapse, hypermobility syndrome (Ehlers-Danlos type 3), irritable bowel syndrome, chronic fatigue syndrome, and inflammatory bowel disease.[4,13] In addition to the usual preoperative concerns related to the surgical procedure, additional preoperative information includes factors which may precipitate the POTS crisis as well as the current medical therapy for the disorder. Continuing these medications during the perioperative period with the administration of these medications the morning of surgery is recommended. In most cases, intravenous preparations can be substituted when oral administration is not feasible. In our patient, perioperative therapy with both an intravenous form of α-adrenergic agonist (phenylephrine) and a β-adrenergic antagonist (metoprolol) were administered.

Intraoperatively, the primary concern of the anesthesiologist, when caring for patients with POTS, is the potential for exaggerated hemodynamic instability, especially during periods of relative hypovolemia. In the normal state, alterations in blood pressure due to hypovolemia are generally corrected by reflex vasoconstriction thereby maintaining blood pressure. As the primary defect in POTS is failure of this compensatory mechanism, fluctuations in blood pressure may occur with relatively minimal changes in intravascular status. Tachycardia associated with POTS has been shown to respond acutely to the bolus administration of isotonic fluids to correct the underlying hypovolemia.[18] In our patient, given the potential for fluctuations in intravascular volume and blood loss during posterior spinal fusion, a central venous cannula was placed to monitor central venous pressure as a guide for fluid replacement and the CVP maintained at 8-10 mmHg with the administration of isotonic fluids or colloid.

Additionally, an arterial cannula was placed for continuous blood pressure monitoring. Although routinely placed during anesthetic care for posterior spinal fusion, invasive arterial and central venous pressure monitoring may also be indicated for less invasive surgical procedures in patients with POTS given the potential for hemodynamic instability. Additionally, when feasible, anesthetic agents that cause clinically significant arterial and venous vasodilatation (such as isoflurane and desflurane) should be avoided. Agents that result in tachycardia or have anticholinergic side effects (ketamine, pancuronium, glycopyrrolate) should be avoided. In the hyperadrenergic sub-type, additional concerns include avoidance of agents that stimulate the sympathetic nervous system or result in catecholamine release (desflurane, ketamine). Care is suggested with the administration of agents that may provoke histamine release (atracurium, mivacurium) and its resultant vasodilatory properties. Given its lack of effects on the vasculature, etomidate may be a prudent choice when intravenous induction is chosen.[19] Given our patient′s preference, anesthetic induction included an inhalation induction using sevoflurane followed by placement of an intravenous cannula. Following anesthetic induction, initial maintenance anesthesia during placement of arterial and venous cannulae included sevoflurane. Given the use of both motor and somatosensory evoked potential monitoring in our patient, a total intravenous anesthetic was used during the procedure which included a combination of propofol and remifentanil. Although our usual practice is to include dexmedetomidine in such cases as a means to limit intraoperative propofol requirements and facilitate awakening, given its sympatholytic effect, it was not used in this case.[20,21] Intraoperatively, occasional bolus doses of phenylephrine were needed to treat periodic episodes of hypotension while metoprolol was used to control heart rate.

There are limited data on which to base decisions regarding the choice of neuromuscular blocking agents in these patients. Although many patients with POTS may manifest chronic fatigue and may not tolerate the normal activities of daily living, there are no data to suggest direct involvement of skeletal muscle which would alter the response to neuromuscular blocking agents. However, when neuromuscular blocking agents are necessary, it may be prudent to use short-acting agents such as cisatracurium, which lack hemodynamic effects. In patients with severe fatigue who are bed ridden, avoidance of succinylcholine is suggested given the potential for an exaggerated hyperkalemic response in patients who are immobile.[22]

The POTS is an autonomic disorder characterized by tachycardia when assuming the upright position. Of primary concern to the anesthesiologist in the perioperative management of patients with POTS is the potential for hemodynamic instability related either to the primary disease process or the failure of the normal sympathetic response when vasodilatation occurs in response to anesthetic agents. An understanding of the pathophysiology of the disease, choice of anesthetic agents with minimal effects on hemodynamic function, and appropriate invasive monitoring of hemodynamic status are suggested to minimize the potential hemodynamic instability during the provision of general anesthesia in this unique patient population.
